# Mutant calreticulin causes essential thrombocythemia

**DOI:** 10.18632/oncotarget.21292

**Published:** 2017-09-28

**Authors:** Kazuya Shimoda, Kotaro Shide, Takuro Kameda

**Affiliations:** Kazuya Shimoda: Department of Gastroenterology and Hematology, Faculty of Medicine, University of Miyazaki, Miyazaki, Japan

**Keywords:** CALR, JAK2

Polycythemia vera (PV), essential thrombocythemia (ET), and primary myelofibrosis (PMF) are categorized as myeloproliferative neoplasms (MPNs). All are characterized by the autonomous growth of one or more lineages of hematopoietic cells, splenomegaly, constitutional symptoms such as fatigue, night sweating, itching, and weight loss, and is frequently complicated by thrombosis and hemorrhage. *JAK2* mutations are observed in more than 95% of PV patients, and about half of ET and PMF patients. About two-thirds of *JAK2* mutation-negative ET and PMF patients harbor *Calreticulin* (*CALR*) mutations. *CALR* mutations with a 52-bp deletion or a 5-bp insertion in exon 9 occur in more than 80% of patients with *CALR* mutations, and cause frameshifts that result in proteins with novel C-terminal domains composed of many positively charged amino acids [1]. As *CALR* mutations are exclusively observed in conjunction with *JAK2* mutations in MPN patients, mutant CALR is speculated to have a driver role in MPNs.

**Figure 1:**
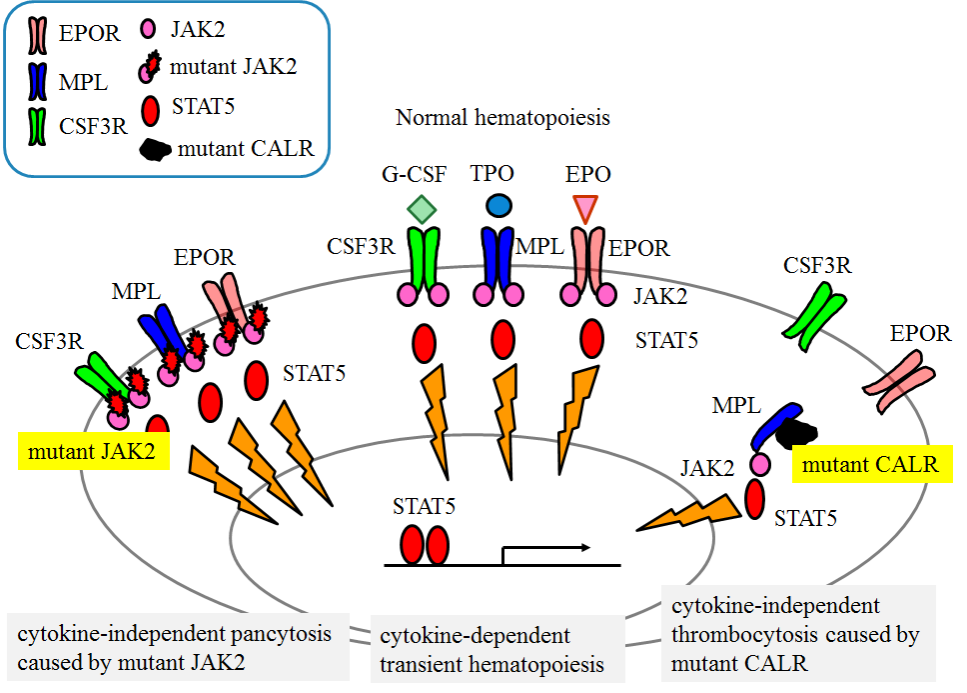
JAK-STAT signaling cascade in normal and MPN hematopoiesis.

Cytokines such as erythropoietin (EPO), thrombopoietin (TPO), and granulocyte colony-stimulating factor bind to their specific cell surface receptors, commonly activate the JAK-STAT signaling cascade, and induce cytokine-dependent transient hematopoiesis [2]. In cases of *JAK2* mutation, JAK2 and STAT5 are constitutively activated without cytokine stimulation, and cause autonomous cell growth (Figure 1). As CALR is best known for its endoplasmic reticulum chaperone functions, assisting in glycoprotein folding, the question has been raised as to why mutant CLAR causes MPN.

We recently reported that in the presence of MPL, mutant CALR augmented the transcriptional activity of STAT5, but not of EPO receptor or CSF3R [3]. When *CALR* mutations are knocked-in human hematopoietic cell lines expressing MPL using the CRISPR-Cas9 system, these cell lines demonstrate increased growth or acquire cytokine-independent cell growth accompanied by STAT5 phosphorylation. These observations indicate that mutant CALR induces cytokine-independent activation of the JAK-STAT signaling cascade in cells expressing MPL, which results in increased cell growth or cytokine-independent cell growth (Figure 1).

In line with these observations, *CALR* mutant mice demonstrate thrombocythemia and develop ET, but do not exhibit erythrocytosis or granulocytosis [3]. This is consistent with many reports in which ET patients with a *CALR* mutation have lower Hb levels and/or leukocyte counts compared to ET patients with a *JAK2* mutation. In addition, ruxolitinib, which is a JAK inhibitor that ameliorates splenomegaly and constitutional symptoms associated with MF, and ameliorates elevated hematocrit values and splenomegaly associated with PV, attenuates the increased numbers of peripheral blood platelets and BM megakaryocytes in *CALR* mutant mice. The effect of ruxolitinib on mice with *CALR* mutations also supports the idea that mutant CALR autonomously activates the JAK-STAT signaling cascade and induces sustained thrombocytosis with increased numbers of megakaryocytes. Our observations, together with previous reports [4-7], indicate that the *CALR* mutation is sufficient to augment megakaryocytic cell growth and cause ET, and that *CALR* mutations, like the *JAK2* mutation, play a driver role in MPNs.

MPL is expressed not only on megakaryocytes, but also on hematopoietic stem cells (HSCs). TPO stimulation is reported to increase HSC numbers *in vitro* and *in vivo.* The proportion of HSCs in BM is higher in *CALR* mutant mice than in WT mice, which is probably due to the constitutional activation of STAT5 by mutant CALR in HSCs. Although the number of HSCs in BM from *CALR* mutant mice is elevated, HSCs with *CALR* mutation do not demonstrate greater self-renewal activity than WT HSCs. In the first recipients in serial competitive transplantation assays, BM cells with a *CALR* mutation exhibit a growth advantage compared to WT cells. In the second recipients, BM cells with a *CALR* mutation exhibit almost the same growth as WT BM cells. This also occurs in *JAK2* mutated-HSCs, suggesting that other kinds of mutations in addition to those of *CALR* and *JAK2* may be required for the full development of MPNs [8].

Mutant CALR plays a driver role in MPNs, however the precise mechanism whereby mutant CALR activates STAT5 has not been fully clarified. Araki et al. reported that mutant CALR, but not WT CALR, binds to MPL, and that the novel C-terminal domains of the mutant protein are required for this interaction [6]. Elf et al. reported that the transforming activity of mutant CALR is not contained within specific residues within the mutant CALR C-terminus, but the oncogenicity of this mutant is dependent on the positive electrostatic charge of its C-terminus [7]. We generated a murine *CALR* mutation knock-in mice via the deletion of base pairs in the murine CALR C-terminal (submitted manuscript, Shide K et al.). This murine mutant CALR possesses many positively charged amino acids, similar to the human mutant CALR, but the precise amino acid sequences are different from their human counterpart. Murine *CALR* mutation knock-in mice do not develop ET, which suggests that newly generated amino acids in human CALR may be essential in the development of ET.
